# STING mediates nuclear PD-L1 targeting-induced senescence in cancer cells

**DOI:** 10.1038/s41419-022-05217-6

**Published:** 2022-09-15

**Authors:** Je-Jung Lee, So Young Kim, Songhee H. Kim, Seoyeon Choi, Bin Lee, Jeon-Soo Shin

**Affiliations:** 1 Department of Microbiology, Yonsei University College of Medicine, Seoul, South Korea; 2 Institute for Immunology and Immunological Diseases, Yonsei University College of Medicine, Seoul, South Korea; 3 Brain Korea 21 FOUR Project for Medical Science, Yonsei University College of Medicine, Seoul, South Korea; 4 Center for Nanomedicine, Institute for Basic Science (IBS), Yonsei University, Seoul, South Korea

**Keywords:** Cancer immunotherapy, Senescence

## Abstract

Immune checkpoint molecule programmed death-ligand 1 (PD-L1) is overexpressed in cancer cells and imparts resistance to cancer therapy. Although membrane PD-L1 has been targeted for cancer immune therapy, nuclear PD-L1 was reported to confer cancer resistance. Therefore, it is important to regulate the nuclear PD-L1. The mechanisms underlying the therapeutic efficacy of PD-L1 targeting have not been well-established. Cellular senescence has been considered a pivotal mechanism to prevent cancer progression, and recently, PD-L1 inhibition was shown to be involved in cancer cell senescence. However, the relevance of PD-L1 targeting-induced senescence and the role of stimulator of interferon genes (STING) has not been reported. Therefore, we aimed to identify the role of PD-L1 in cancer progression and how it regulates cancer prevention. In this study, we found that PD-L1 depletion-induced senescence via strong induction of STING expression in mouse melanoma B16-F10 and colon cancer CT26 cells, and in human melanoma A375 and lung cancer A549 cells. Interestingly, nuclear PD-L1 silencing increased STING promoter activity, implying that PD-L1 negatively regulates STING expression via transcriptional modulation. Furthermore, we showed that PD-L1 binds to the STING promoter region, indicating that PD-L1 directly controls STING expression to promote cancer growth. In addition, when we combined PD-L1 silencing with the senescence-inducing chemotherapeutic agent doxorubicin, the effect of PD-L1-targeting was even more powerful. Overall, our findings can contribute to the understanding of the role of PD-L1 in cancer therapy by elucidating a novel mechanism for PD-L1 targeting in cancer cells.

## Introduction

Cellular senescence entails permanent cell cycle arrest and can be induced by various stress stimuli, including DNA damage, oncogene activation, telomere dysfunction, and reactive oxygen species buildup [1]. Senescent cells are characterized by morphological changes, positive senescence-associated β-galactosidase (SA-β-Gal) activity, increased expression of cyclin-dependent kinase (CDK) inhibitors such as p16, p21, and p27, secretion of several inflammatory factors called the senescence-associated secretory phenotype (SASP) [2–4], and the expression of markers related to DNA damage and heterochromatin like γ-H2AX and H3K9 trimethylation [5–7]. Cellular senescence can help to suppress tumor growth by interrupting the cell cycle and proliferation of cells resistant to apoptosis following cancer therapy [7–12].

Programmed death-ligand 1 (PD-L1), also known as B7 homolog 1 (B7-H1) or CD274, is a major obstacle to antitumor immunity because it confers resistance to tumor-reactive T cells when bound to its receptor PD-1 (CD279). PD-L1 is expressed abundantly in many malignant cells as well as in vascular endothelial cells, which are located in not only the membrane but also the nucleus. However, mostly membrane PD-L1 has been targeted, which has been limited to the treatment of some cancers [13–15]. Additionally, it was shown to be involved in cancer senescence via interferon-dependent cell cycle regulator pathways or through its destabilization by cyclin D-CDK4 kinase [16–20].

Cyclic GMP-AMP synthase (cGAS)-stimulator of interferon genes (STING) plays a pivotal role in the innate immune pathways via recognizing and responding to the cytosolic DNA. cGAS recognizes and binds to cytoplasmic DNA and recruits STING for downstream reactions. The cGAS-STING pathway is involved in diverse pathological conditions such as apoptosis and senescence [21–25]. In particular, STING induces senescence in cancer via activated NF-κB signaling cascade or SASP secretion. Furthermore, we previously demonstrated that STING is required for HMGB1-mediated-senescence [26].

In the current study, we reported that silencing PD-L1 in cancer cells induced senescence. While strategies for preventing tumor development have been established, for example, via targeting membrane PD-L1 or STING pathway activation, their relevance in inhibiting tumor growth remains unknown, especially via senescence induction. Therefore, in the present study, we aimed to explore the role of STING in nuclear PD-L1-silencing-induced senescence and cancer prevention. We found that nuclear PD-L1-depletion-induced cancer cell senescence via upregulating STING transcription and activating p21, collectively contributed to tumor regression. Furthermore, the effect was more significant in the presence of treatment with doxorubicin (Dox), a chemotherapeutic agent.

## Materials and methods

### Cell culture, transfection, and reagents

B16-F10 mouse melanoma cells and MEF cells were cultured in Dulbecco’s Modified Eagle’s medium. CT26 parent and PD-L1 knockout (KO) mouse colon cancer cells (kindly gifted by Dr. Sang-Jun Ha from Yonsei University, Seoul, Republic of Korea), A549 human lung cancer cells, and Jurkat human T lymphocytes were grown in RPMI-1640. All media were supplemented with 10% fetal bovine serum and 1% penicillin-streptomycin (Life Technologies, Waltham, MA, USA). The PD-L1 plasmid (MG50010-UT) was obtained from Sino Biological (Wayne, PA, USA). Plasmids and siRNA transfections were performed using FuGene HD reagent (Promega, Madison, WI, USA) and RNAiMAX (Invitrogen, Carlsbad, CA, USA), respectively, as recommended by the manufacturers. siRNA duplexes against human and mouse PD-L1, STING, and nonspecific control siRNA were purchased from Bioneer, Inc. (Daejeon, Korea). Dox was obtained from Calbiochem (San Diego, CA, USA). C-178 (S6667) and Santacruzamate A (CAY10683) were purchased from Selleck Chemicals (Houston, TX, USA). A silver staining kit (EBP-1051) was purchased from ELPIS BIOTECH, Inc. (Daejeon, Korea).

### Cell counting

Trypan blue solution (0.4%) was added to the cell suspension and incubated for 5 min at room temperature. Cells lacking staining were counted as viable cells using a hemocytometer under a microscope [27].

### WB analysis

Cells were collected and lysed in RIPA buffer, and the protein concentrations were quantified. Equal amounts of total protein were separated by electrophoresis and transferred onto nitrocellulose membranes. Proteins were detected using specific antibodies. Horseradish peroxidase (HRP)-conjugated anti-mouse and anti-rabbit antibodies were purchased from Santa Cruz Biotechnology (Dallas, TX, USA). Antibodies against PD-L1 (17952-1-1; Proteintech, Rosemont, IL, USA), p16 (ab51243; Abcam, Cambridge, MA, USA), p21 (556431; BD Biosciences, Franklin Lakes, NJ, USA), and p27 (610242; BD Biosciences), STING (13647), tri-H3K9 (13969), pRB (S807/811), Caspase 3 (9662 S), and β-actin (4967 S) were used, and secondary antibodies for immunofluorescence were obtained from Cell Signaling Technologies, Inc. (Danvers, MA, USA). Antibody-antigen complexes were detected using HRP-conjugated secondary antibodies and visualized using a standard chemiluminescence method according to the manufacturer’s instructions.

### Cell morphology analysis and SA-β-Gal staining

Morphological changes in the cells were photographed using an inverted phase-contrast microscope (Olympus, Tokyo, Japan). SA-β-Gal staining was performed as previously described [28]. Morphological examinations were performed on day 3 following each treatment unless otherwise indicated.

### Chromatin immunoprecipitation (ChIP) analysis

Cells were grown to 90% confluence in two 150 cm^2^ dishes per sample. Next, the cells were crosslinked with 0.75% formaldehyde (FA) at 25 °C for 10 min and glycine was added to a final concentration of 125 mM. The samples were centrifuged, washed with PBS, resuspended in lysis buffer [1% SDS, 10 mM ethylenediaminetetraacetic acid (EDTA), 50 mM Tris-HCl (pH 8.1)], and sonicated. The supernatants were then recovered by centrifugation at 12,000 rpm for 10 min at 4 °C and diluted with dilution buffer [0.5% Triton X-100, 2 mM EDTA, 100 mM NaCl, 20 mM Tris-HCl (pH 8.1)]. Next, a 50 μL aliquot of purified DNA was used to calculate the DNA concentration. Chromatin amount equivalent to ~25 μg DNA was used for each IP, and 2 μg anti-PD-L1 antibody with 20 μL protein G beads (50% slurry) was combined and incubated overnight. Complexes were eluted by adding 250 μL elution buffer (1% SDS/0.1 M NaHCO_3_) to pelleted beads for reverse crosslinking and centrifuged. Pelleted DNA from the supernatants was purified using a spin column, and PCR was performed using target-specific primers.

### DNA pull-down assay

The STING promoter DNA (from −1154 to −624 bp) was obtained by performing PCR with target-specific biotinylated primers and mixed with streptavidin-conjugated magnetic beads. Crosslinking with FA was carried out after the addition of untreated or Dox-treated B16-F10 cell lysates, and immunoprecipitation was performed. We washed the cells with buffers containing low salt concentrations (#1) to high salt concentrations (#6) for eluting specific DNA-binding proteins. The eluted proteins were identified using silver staining and western blotting.

### Luciferase reporter assay

Cells were seeded onto 12-well plates and co-transfected with a firefly luciferase reporter fused to the *STING* promoter (100 ng) and either an empty vector or a vector expressing *STING*, siRNA control (SiC), or siRNA targeting PD-L1 (SiPD-L1). Three days after transfection, the lysates were analyzed for luciferase reporter activity (Promega, Madison, WI, USA), according to the manufacturer’s protocol.

### Immunofluorescence

Cells were cultured in four-well glass slides (Lab-Tek II Chamber Slide; Thermo Fisher Scientific) treated with poly l-lysine solution (P4787; Sigma) and fixed with 4% paraformaldehyde solution after treatment. After permeabilization with 1% Triton X-100, cells were stained using appropriate antibodies and a fluorochrome-tagged secondary antibody. The slides were mounted with DAPI (Vector Laboratories, Burlingame, CA, USA) and observed under an FV1000 confocal microscope (ZEISS, LSM700, Jena, Germany).

### Mouse experiment

All animal procedures were approved by the Institutional Animal Care and Use Committee (IRB no. 2019-0242). Briefly, 7–8-week-old female BALB/c mice were housed in a specific pathogen-free facility and used for allograft tumor experiments. To generate tumors, 1 × 10^6^ CT26 cells suspended in 100 μL PBS were injected into the dorsal subcutaneous area of the mice, and tumor masses were successfully formed after implantation. For Dox treatment, mice were administered a single intraperitoneal injection of Dox (9 mg/kg body weight) after tumor formation, and the tumors were collected 7 days later. Tumor tissues were fixed overnight in 4% formalin, embedded in paraffin, and subjected to immunohistochemistry to detect STING and H3K9me3 expression. The sections were counterstained with DAPI. Photographs were acquired in randomly chosen fields per tumor section according to standard procedures.

### Statistical analysis

Statistical analysis was performed using GraphPad Prism 5 (GraphPad Software, Inc., La Jolla, CA, USA). The significance of the statistical differences among three or more groups was calculated using a one-way analysis of variance and the Newman–Keuls test. Data are shown as the mean ± standard deviation (SD). Asterisks denote the *p*-values as follows: *p < 0.05, **p < 0.01, and ***p < 0.001.

## Results

### Targeting PD-L1 causes cellular senescence in cancer cells

**Fig. 1:**
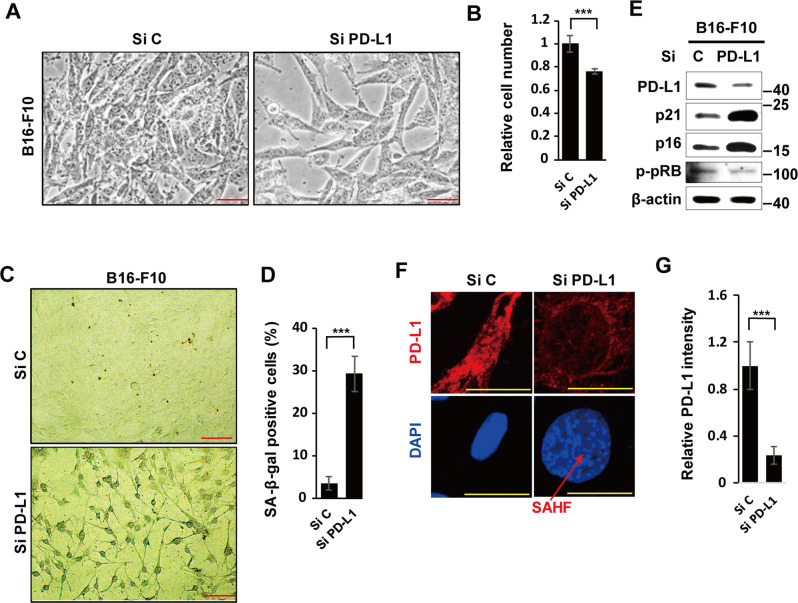
Targeting PD-L1 induces cellular senescence in cancer cells. B16-F10 cells were transfected with 100 nM control (siC) or PD-L1 (siPD-L1) siRNA. The morphological changes (**A**), relative cell numbers (**B**), and SA-β-Gal-positive cells were counted from 100 cells (**C**, **D**), and western blot analysis of the indicated proteins (**E**) was performed on day 3 after siRNA treatment. β-Actin was used as the loading control. In addition, PD-L1 and nuclei were stained, and senescence-associated heterochromatin foci (SAHF) are shown by DAPI staining in SiPD-L1-treated cells (**F**). The PD-L1 intensity from 100 cells was measured using the ImageJ program (**G**). Scale bars = 50 μm (**A**, **C**) and 20 μm (**F**). Statistical significance was determined by an unpaired, two-tailed Student’s *t* test with *P* < 0.05 was performed. Quantitative data are expressed as means ± SD. *n* = 3, ***P < 0.001. NS not significant.

Although targeting PD-1/PD-L1 has been successful in some cancers, its low efficacy is a common concern. Hence, a better understanding of the regulatory mechanisms underlying the role played by PD-L1 is critical to overcoming this limitation. The nuclear location of PD-L1 suggests that it plays a key role in regulating tumors [29]. Here, we silenced PD-L1 expression in B16-F10 mouse melanoma cells using siRNA and observed an increased cell size (Fig. 1A), decreased cell proliferation (Fig. 1B), and increased SA-β-Gal positivity (Fig. 1C, D) compared with control siRNA treated cells. We then checked whether these effects were related to the senescence phenotype by evaluating protein expression of the CDK inhibitors p21 and p16, and the tumor suppressor phospho-pRB (p-pRB). Upregulated CDK inhibitors, p21, and p16 inhibited p-pRB expression via blocking the CDK, which is a characteristic feature of senescence (Fig. 1E). In addition, senescence-associated heterochromatin foci (SAHF), which is also senescence marker, was observed in PD-L1-silenced senescent cells (Fig. 1F, G). Collectively, these results indicate that PD-L1 deficiency-induced senescence in B16-F10 cancer cells.

### PD-L1 depletion accelerates Dox-induced senescence

**Fig. 2:**
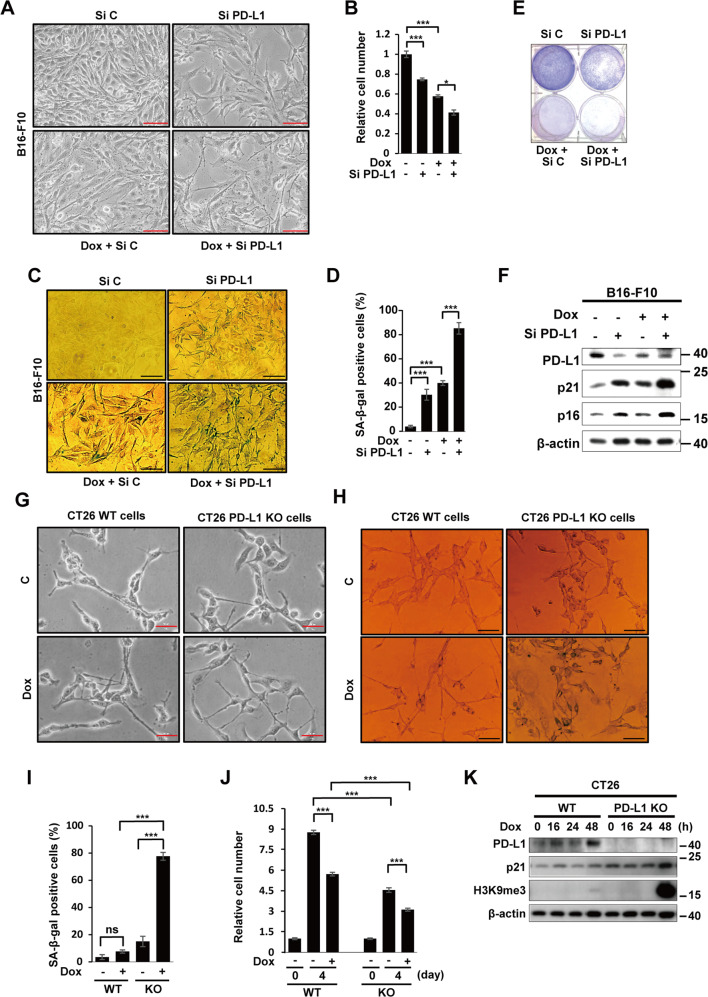
PD-L1 depletion accelerates Dox-induced senescence. **A**–**F** B16-F10 cells were transfected with 100 nM Control (siC) or PD-L1 (siPD-L1) siRNA prior to treatment with 100 ng/mL Dox. The morphological changes (**A**), relative cell numbers (**B**), and SA-β-Gal-positive cells were evaluated in 100 cells (**C**, **D**). The colony-forming assay was performed on day 7 (**E**) and western blot analysis (**F**) of the indicated proteins was performed on day 3 post-Dox treatment. Parent and PD-L1 KO CT26 cells were treated with 100 ng/mL Dox for 3 days (**G**–**K**) and imaged under a phase-contrast microscope (**G**). SA-β-Gal-positive cells from 100 cells (**H**, **I**) were counted. Relative cell numbers were quantified (**J**) on days 0 and 4 after Dox treatment, and western blotting was performed at the indicated time points after treatment (**K**). β-actin was detected as a loading control. Scale bars = 50 μm (**A**, **C**, **G**, **H**). The significance of the statistical difference among three group was calculated using a one-way analysis of variance and Newman-Keuls. Quantitative data are expressed as means ± SD. ns, not significant. *P < 0.05. ***P < 0.001.

Senescence can be induced by low doses of Dox treatment in cancer cells [26, 30–33]. In this study, Dox-treated B16-F10 cells underwent senescence characterized by typical senescent morphology and relative cell number (Fig. 2A, B), positive staining for SA-β-Gal (Fig. 2C, D), lower colony-forming efficacy (Fig. 2E), and increased expression of CDK inhibitors p16 and p21 (Fig. 2F). All of these effects were accelerated by PD-L1 targeting with siRNA.

We next treated wild-type (WT) and PD-L1 KO CT26 mouse colon cancer cell lines with Dox. As expected, only Dox-treated-PD-L1 KO CT26 cells showed larger and flattened senescence-like morphology (Fig. 2G) and positive staining for SA-β-Gal (Fig. 2H, I). The growth rate for untreated KO cells was much slower than that for untreated WT cells at 4 days, while the growth of both cell types was suppressed under Dox treatment (Fig. 2J). Additionally, PD-L1 KO cells showed increased H3K9me3 and p21 protein expression after Dox treatment (Fig. 2K). Taken together, we conclude that depletion of PD-L1 enhanced the inhibition effects of tumor cell growth via promoting senescence induction by Dox treatment.

### STING mediates PD-L1 targeting-induced senescence

**Fig. 3:**
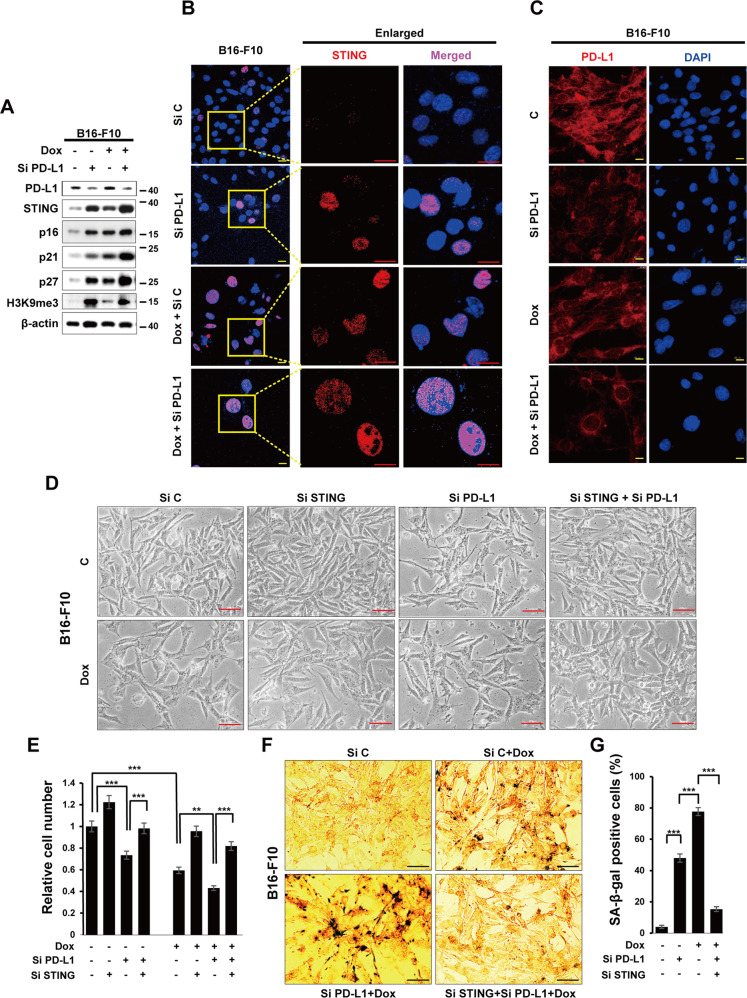
STING mediates PD-L1 targeting-induced senescence. **A**–**C** B16-F10 cells were transfected with 100 nM Control (siC) or PD-L1 (siPD-L1) siRNA prior to treatment with 100 ng/mL Dox. Western blot analysis of the indicated proteins was performed on day 3 after Dox treatment (**A**). Endogenous STING and PD-L1 expression were determined by confocal microscopy following staining with anti-STING or anti-PD-L1 antibodies with the appropriate secondary antibodies on day 3 after Dox treatment (**B**, **C**). Scale bars = 20 μm. **D**–**G** B16-F10 cells were individually transfected with 100 nM siRNAs against STING (siSTING), Control (siC), or PD-L1 (siPD-L1). Then, cells were treated with 100 ng/mL Dox and the morphological changes (**D**), relative cell numbers (**E**), and SA-β-Gal-positive cells (**F**, **G**) were analyzed on day 3 post treatment. β-actin was detected as a loading control. Scale bars = 20 μm (**B**, **C**) and 50 μm (**D**, **F**). The significance of the statistical difference among the three group was calculated using a one-way analysis of variance and Newman–Keuls. Quantitative data are expressed as the mean ± SD. **p < 0.01, ***p < 0.001.

We investigated the mechanism by which PD-L1 downregulation led to senescence. Recent studies have shown that STING is involved in cellular senescence (Dou et al., 2017) and the synergistic effects of PD-L1 and STING in tumor suppression [34, 35]. However, it has not been studied about the involvement of the role of STING signaling in PD-L1 pathway. Therefore, we examined whether STING plays a role in PD-L1 targeting-induced senescence. First, we silenced PD-L1 expression in B16-F10 cells and investigated STING expression. Interestingly, STING protein expression was significantly increased by PD-L1 silencing, which was accompanied by other senescence markers p16, p21, p27, and H3K9me3 by an individual or dual treatment with siPD-L1 and Dox, as shown by western blot analysis (WB) (Fig. 3A), immunofluorescence assay (Fig. 3B, C), and SA-β-Gal staining (Fig. S1A–D). These results were further confirmed in A375 human skin cancer cells (Fig. S2A–D) and A549 human lung cancer cells (Fig. S2E, F), as well as non-cancerous cells such as mouse embryonic fibroblasts (MEF) (Fig. S3A, B) and Jurkat human T cells (Fig. S3C, D). Next, we pre-treated B16-F10 cells with STING siRNA to verify the involvement of STING in PD-L1 deficiency-induced senescence. Silencing STING reversed the changes in morphology (Fig. 3D) and cell number (Fig. 3E), and SA-β-Gal staining (Fig. 3F, G) induced by PD-L1 depletion. Additionally, micronuclei were induced by SiPD-L1 treatment and even more increased in Dox treatment together (Fig. S4A), and the manner is consistent with the degree of SA-β-Gal activity (Fig. S4B). SASP is required for activation of STING pathway. IL-6 and IL-8 expression were also upregulated in SiPD-L1 treated group and more increased by Dox treatment (Fig S4C). To confirm that STING pathway is required for PD-L1 depletion-induced senescence, we employed STING inhibitor C-178, which binds to Cys91 on STING to block its palmitoylation and prevent recruitment and phosphorylation of TBK1. As expected, the STING inhibitor, C-178 prevented cell enlargement and the canonical STING pathway, including p-TBK and p-IRF (Fig. S4D, E). Therefore, C-178 rescued the effects of PD-L1 depletion on B6-F10 cell senescence. Consequently, all those STING pathways can be fully activated by elevated STING expression induced by PD-L1 depletion. Altogether, these data indicate that depletion of PD-L1 induces senescence via modulation of the STING pathway.

Additionally, when we overexpressed PD-L1, all those effects from PD-L1 depletion were reversed. In other words, PD-L1 overexpression abolished the Dox-induced senescence phenotype, including loss of morphological changes, reduced protein expression, and increased propidium iodide (PI) positivity in B16-F10 (Fig. S5A–C) and PD-L1 KO CT26 (Fig. S5D–F) cells. Furthermore, morphological features of senescence induced by Dox in B16-F10 cells were prevented (Fig. S5A) and the expression of p21, p16, H3K9me3, and STING was diminished by PD-L1 overexpression (Fig. S5B). Moreover, PD-L1 overexpression led to apoptosis in both cell types, which was confirmed by caspase 3 activation and PI staining positivity (Fig. S5B–C, E–F). In conclusion, PD-L1 overexpression not only abolished Dox-induced senescence but also induced apoptosis. Previous studies have also shown an alternative cell fate between senescence and apoptosis, depending on the presence of target factors [11, 30].

Based on these findings, we conclude that STING is necessary for PD-L1 targeting-induced senescence of cancer cells.

### Abrogating nuclear PD-L1 induces senescence via STING upregulation

**Fig. 4:**
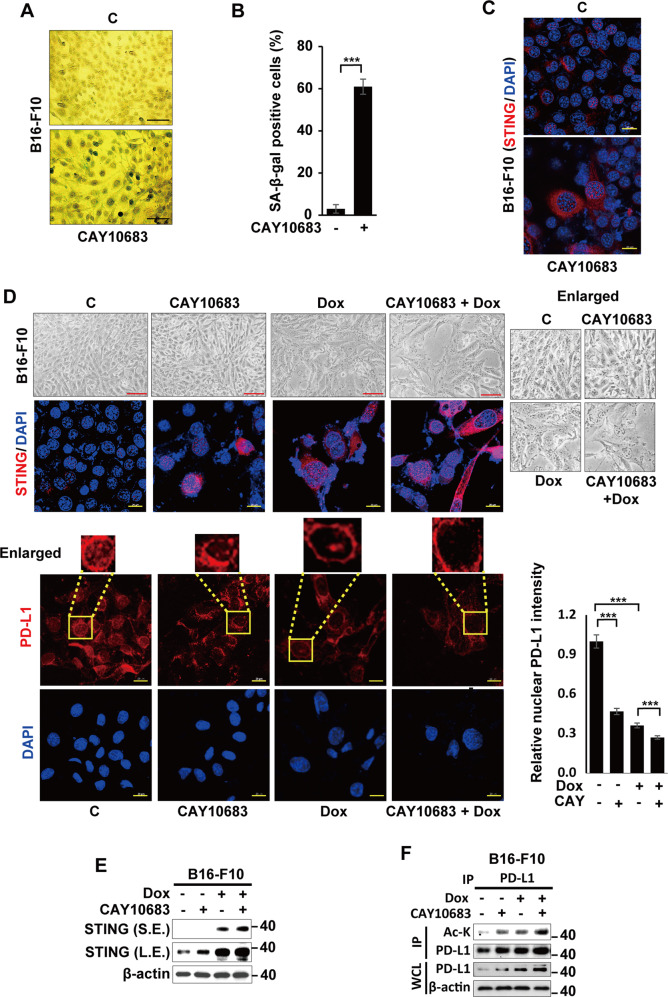
Suppressing nuclear PD-L1 induces senescence via STING upregulation. B16-F10 cells were treated with pharmacological HDAC2 inhibitor (CAY10683). The SA-β-Gal-positive cells were photographed (**A**) and enumerated (**B**), and STING expression was analyzed by an immunofluorescence assay (**C**) on day 3 post-treatment. B16-F10 cells were treated with HDAC2 inhibitor 6 h prior to treatment with 100 ng/mL Dox, and imaged on day 3 post-treatment; STING expression and PD-L1 localization were analyzed by an immunofluorescence assay. Relative nuclear PD-L1 intensity was analyzed using ImageJ (*n* = 100) (**D**). Western blot analysis was performed (**E**) and PD-L1 acetylation was demonstrated using an anti-pan acetyl PD-L1 antibody on day 3 post-treatment (**F**). (S.E. short exposure, L.E. long exposure). Scale bars = 50 μm (**A**, **D**; upper panel) and 20 μm (**C**, **D**; lower panel). Statistical significance was determined by an unpaired, two-tailed Student’s *t* test with *P* < 0.05 (**B**) or one-way analysis of variance and Newman–Keuls (**D**). Quantitative data are expressed as the mean ± SD. ***p < 0.001.

Cells undergoing senescence following Dox treatment as well as PD-L1-silencing showed significantly decreased PD-L1 expression in the nuclei compared to control cells (Figs. 1F, 3C). Recently, different functions of PD-L1 depending on its cellular localization were reported [36–38]. Therefore, based on our results, we also investigated if the abrogating PD-L1 expression in the nucleus is a prerequisite for senescence induction. We used CAY10683, a known HDAC2 inhibitor, to force the export of PD-L1 from the nucleus by acetylation. This disappearance of PD-L1 in the nucleus led to less malignant cancer [29]. Indeed, CAY10683 treatment induced significant senescence in B16-F10 cells (Fig. 4A–C) and also promoted Dox-induced senescence (Fig. 4D–F), as evident by an increase in SA-β-Gal positivity (Fig. 4A, B), cell size, and STING expression (Fig. 4C–E). Simultaneously, PD-L1 acetylation by CAY10683 (Fig. 4F) led to PD-L1 disappearance from the nucleus (lower panel in Fig. 4D). These observations indicated that abrogating nuclear PD-L1 expression is necessary for the senescence induction in cancer cells.

### PD-L1 regulates STING transcription

Based on our results (Fig. 4), we next investigated how PD-L1 regulates STING expression. We constructed a pGL3 luciferase reporter plasmid combining mouse STING promoter sequences (pGL3-mSTING-luc) and checked for STING promoter activity.

**Fig. 5:**
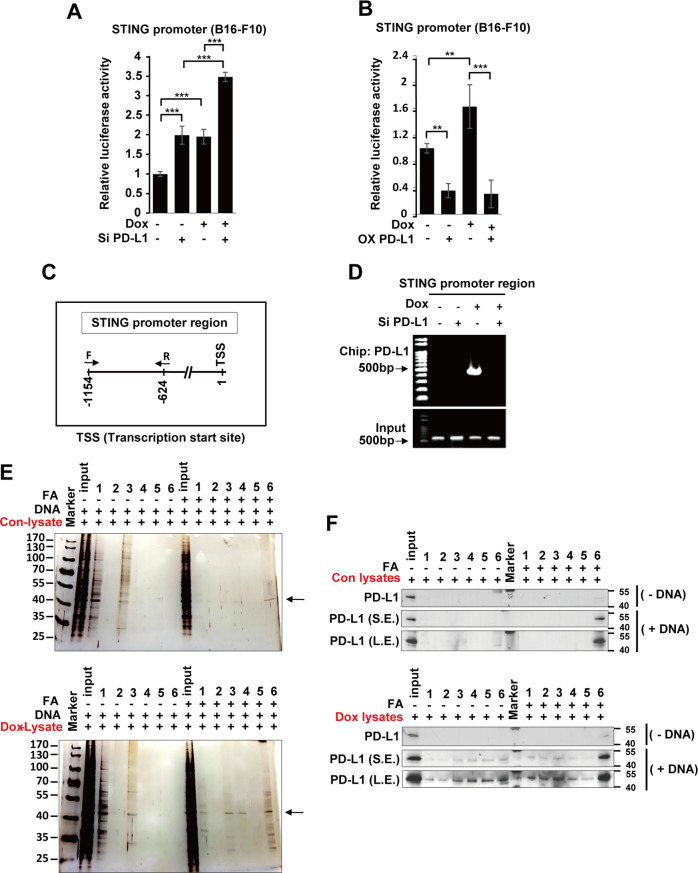
PD-L1 modulates STING transcription. B16-F10 cells were transfected with the STING promoter harboring plasmid prior to siRNA treatment or PD-L1 overexpression. Then, cells were treated with 100 ng/mL Dox and a luciferase assay for STING promoter activity was performed on day 3 post-treatment (**A**, **B**). Additionally, B16-F10 cells were transfected with 100 nM control (siC) or PD-L1 (siPD-L1) siRNA prior to treatment with 100 ng/mL Dox, and an immunoprecipitation assay (ChIP) was performed with an anti-PD-L1 antibody. PCR was performed using the indicated STING promoter-specific primers for ChIP assays (**C**, **D**). For the DNA pull-down assay, specific PD-L1 bands bound to the STING promoter DNA (from −1154 to −624 bp) were eluted with the indicated washing buffer (1:150 mM NaCl-1st washing; 2: 150 mM NaCl-2nd washing; 3: 250 mM NaCl-1st washing; 4: 250 mM NaCl-2nd washing; 5: 500 mM NaCl-1st washing; 6:500 mM NaCl-2nd washing). Finally, the results were analyzed by silver staining and western blotting, respectively (**E**, **F**). Arrows indicate the PD-L1 protein. FA: formaldehyde, *S.E.* short exposure, *L.E.* long exposure. The significance of the statistical difference among the three groups was calculated using a one-way analysis of variance and Newman-Keuls. Quantitative data are expressed as the mean ± SD. **p < 0.01, ***p < 0.001.

B16-F10 cells were overexpressed with pGL3-mSTING-luc and treated with Dox and siPD-L1, or overexpressing PD-L1 either. Luciferase activity under the STING promoter was significantly elevated in either siPD-L1 or Dox treatment, and aggravated by co-treatment of Dox and siPD-L1 (Fig. 5A). However, luciferase activity under the STING promoter was decreased by PD-L1 overexpression with or without Dox -treatment (Fig. 5B). We then investigated if PD-L1 binds to STING promoter region through a ChIP assay. We designed primers targeting the STING promoter regions (Fig. 5C) and performed the ChIP assay. Interestingly, PD-L1 showed significant binding to the STING promoter region in the Dox treatment condition; however, the binding was abrogated in PD-L1 knockdown condition in B16-F10 cells (Fig. 5D). Additionally, a DNA pull-down assay was performed wherein STING promoter DNA (from −1154 to −624 bp) bound PD-L1 was precipitated from control and Dox-treated B16-F10 cell lysates, and specific PD-L1 bands were detected by both silver staining and western blotting. Following serial elution with low salt (#1) to high salt buffers (#6), specific PD-L1 bands were detected in the high salt buffer (500 mM NaCl) by both analyses. Additionally, crosslinking with FA enhanced the PD-L1 band. PD-L1 band in the Dox-treated lysates was more intense than that in the control lysates (Fig. 5E, F), which may be attributed to the effect of Dox on DNA torsion and chromatin dynamics that enhances PD-L1 interaction with STING promoter [39]. Taken together, these results demonstrate that PD-L1 regulates STING transcription by binding to the STING promoter region.

### Abrogation of PD-L1 expression suppresses tumor growth via senescence

**Fig. 6:**
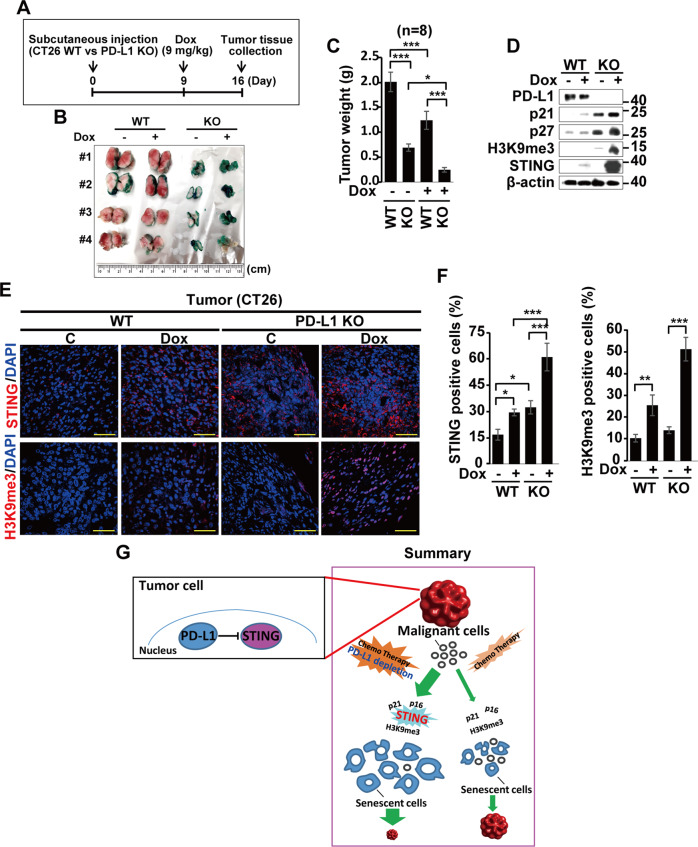
PD-L1 regulates tumor growth via senescence. Tumors were generated by implanting parent or PD-L1 KO CT26 cells into mice (**A**). On day 9 after implantation, mice were administered Dox (9 mg/kg) for seven days, and tumors were excised, cut in half, and analyzed by SA-β-Gal staining (*n* = 4) (**B**). Tumor weights analysis (*n* = 8), (**C**), western blot analysis of tumor cell lysates for the indicated proteins (**D**), and tissue immunostaining for STING and H3K9me3 proteins were performed and quantified (**E**, **F**). Scale bars = 10 μm. (**G**) A model summarizing PD-L1 targeting-induced senescence in tumor cells. While cancer cells can be suppressed by chemotherapy-induced senescence, targeting nuclear PD-L1 accelerates senescence via elevating STING expression, which exerts significant tumor suppression effects together with conventional chemotherapy. Scale bars = 100 μm. The significance of the statistical difference was calculated using a one-way analysis of variance and Newman–Keuls. Quantitative data are expressed as means ± SD. *n* = 8. Quantitative data are expressed as the mean ± SD. *p < 0.05, **p < 0.01, ***p < 0.001.

Finally, we aimed to confirm these results in vivo by constructing mouse tumor models via implanting wild and PD-L1 KO CT26 cancer cells (1 × 10^6^) in both flanks. Nine days later, we injected Dox into the intraperitoneal region, and tumors were collected seven days after Dox treatment (Fig. 6A). Compared with WT-derived tumors, both untreated- and Dox-treated-PD-L1 KO-derived tumors showed strong SA-β-Gal activity, and their sizes were much smaller than those of WT-derived tumors (Fig. 6B). The weights of the tumors were much lower in the KO groups compared to WT groups, as well as in the Dox-treated groups compared to the untreated groups in both WT and KO cells (Fig. 6C). Consistent with the in vitro data (Fig. 2K), WB analysis showed upregulated expression of p21, p27, H3Kme3, and STING in the vehicle treated-PD-L1 KO tumors compared with WT tumors, and these levels were highly elevated in Dox-treated-PD-L1 KO tumors but not in WT tumors (Fig. 6D). Finally, STING and H3K9me3 positivity of Dox-treated KO tumors were much higher than those of Dox-treated WT tumors (Fig. 6E, F). Taken together, our results provide credible evidence that PD-L1 depletion suppresses tumor growth via induction of cellular senescence mediated by STING.

## Discussion

This study demonstrates that PD-L1 silencing contributes to the inhibition of cancer growth via inducing premature senescence. Specifically, blocking PD-L1 binding to the STING promoter upregulates STING expression and leads to the senescence of cancer cells. In contrast to the previous reports on the role of cell membrane PD-L1 in immune function, we elucidated the pivotal role of nuclear PD-L1 in cancer regulation, which suggests that targeting nuclear PD-L1-induced senescence via STING can contribute to full tumor regression. Our finding can be supported by the previous report that PD-L1 can directly regulate gene expression at the transcription level. For example, PD-L1 directly regulates the expression of embryonic stem-cell transcription factors, including Oct-4, Nanog, and the stemness factor BMI1 [40, 41].

While most studies on the PD-1/PD-L1 pathway have focused on the cell surface expression of PD-L1, some studies have reported that intrinsic PD-L1 signaling conferred enhanced tumor cell proliferation and growth in ovarian and melanoma cancer cells [36]. The distribution and role of intracellular PD-L1 may explain why not all ICB cannot fully prevent PD-L1 biological functions and effectively prevent tumor growth. Regarding this, gene silencing may have advantages over antibody blockade in suppressing PD-L1 sources and functions. Therefore, the genetic control of PD-L1 expression in cells is studied, and it is important to find pivotal molecules to target PD-L1 production for improving clinical efficacy [42–44].

Cellular senescence is a well-established mechanism of tumor growth suppression during chemo- or ionizing radiation therapy [11, 12]. In addition, Immune checkpoint molecules such as PD-L1 are involved in senescence [45, 46], and targeting membrane-bound PD-L1 in cancer cells induces senescence via modulation of cell cycle modulating proteins and CDK inhibitors [17]. However, there have been no studies reporting the role of STING in nuclear PD-L1 targeting-induced senescence. Here, we demonstrated a new pathway involving PD-L1 and STING for anticancer effects via inducing senescence. Nuclear PD-L1 targeting caused senescence in cancer cells via upregulating STING expression, and when combined with anticancer drugs such as Dox, the degree of senescence and anticancer effects increased further. This finding implies that STING may be a primary mediator of PD-L1 targeting-induced senescence. Furthermore, we unveiled that PD-L1 directly regulates STING transcription via negative regulation of STING promoter activity. Therefore, the depletion of PD-L1 elevated STING expression and contributed to senescence induction. Our study can be supported by that the pharmacological or genetic regulation of PD-L1 acetylation can abrogate its nuclear translocation, leading to less metastasized lung cancer. Indeed, metastasized cancer cells showed relatively higher nuclear PD-L1 expression compared to the subcutaneous primary tumors, which showed dominant membrane PD-L1 expression [29]. Consistently, when we treated cells with an inhibitor that prevented PD-L1 nuclear translocation, STING upregulation-mediated senescence was enhanced by Dox co-treatment.

Additionally, identifying the role of PD-L1 in other cellular parts and targeting it would be effective to control PD-L1 function. Furthermore, we wondered whether nuclear PD-L1 is implicated in other pathology beyond tumorigenesis, which can be an important topic for future studies. While a previous study demonstrated that the part of Lys residues in the C-tail of PD-L1 is responsible for DNA binding and suggested several binding motifs of PD-L1 to DNA for gene transcription regulation [29], we need to verify the accurate binding motif of PD-L1 to STING promoter DNA in the future study. Apart from being an immune checkpoint molecule expressed on the cell surface, PD-L1 also binds to certain proteins and regulates the expression and function of downstream factors. For example, PD-L1 directly binds to a high-mobility group protein, HMGA1, and promotes the expression of stemness-associated genes such as ALDH1, Bmi1, ABCG2, and Oct-4 via the AKT and MEK/ERK pathways [47, 48]. In the same line, in the future, the relationship between PD-L1 and STING proteins and their roles in cancer needs to be more studied.

Senescence is an alternative anticancer mechanism for apoptosis-resistant cancer cells [11, 30, 49], and we have previously shown that cancer cells choose either senescence or apoptosis depending on the expression of genes such as *AKT* or *HMGB1* [49]. Interestingly, while PD-L1 depletion-induced senescence, however, PD-L1 overexpression caused apoptosis in the current study.

The results of our study are summarized in Fig. 6G. While the tumor was regressed by chemotherapy (Dox) induced senescence mechanism through cell cycle inhibitors or H3K9me3 upregulation, its efficacy was still limited. In intact tumor status, PD-L1 bound to the STING promoter and suppressed its nuclear expression, thereby contributing to tumorigenesis; however, the genetic silencing or blocking of nuclear PD-L1 resulted in significant STING expression and activation of its activity, which accelerated senescence in tumor cells, eventually causing full tumor regression.

Overall, our study shows that depletion of PD-L1 can contribute to tumor prevention through cellular senescence via STING upregulation, which has important implications for understanding and developing better strategies for cancer immunotherapy.

## Supplementary information

Supplementary Information

Supplementary Western Blot data

Reproducibility checklist

## Data Availability

The data that support the findings of this study are available from the corresponding author on reasonable request.
