# Supplementary material for: STING mediates nuclear PD-L1 targeting-induced senescence in cancer cells
Source: Cell Death Dis. 2022 Sep 15;13(9):791. doi: 10.1038/s41419-022-05217-6 (PMC9477807; doi:10.1038/s41419-022-05217-6)
Supplement: Supplementary file 1 — Supplementary Information [file 41419_2022_5217_MOESM1_ESM.docx]

**Supplementary Materials**

**STING mediates nuclear PD-L1 targeting-induced senescence in cancer cells**


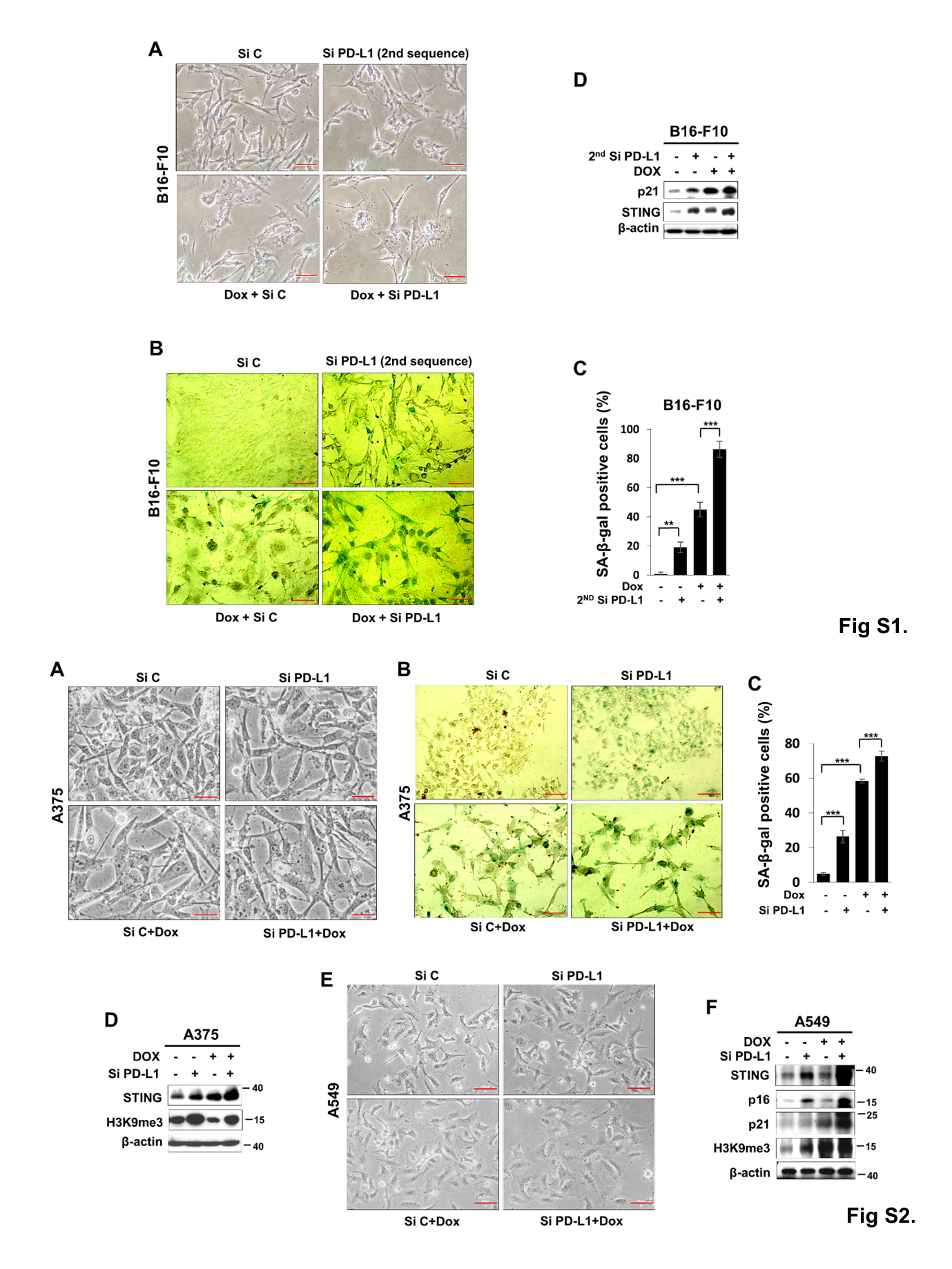


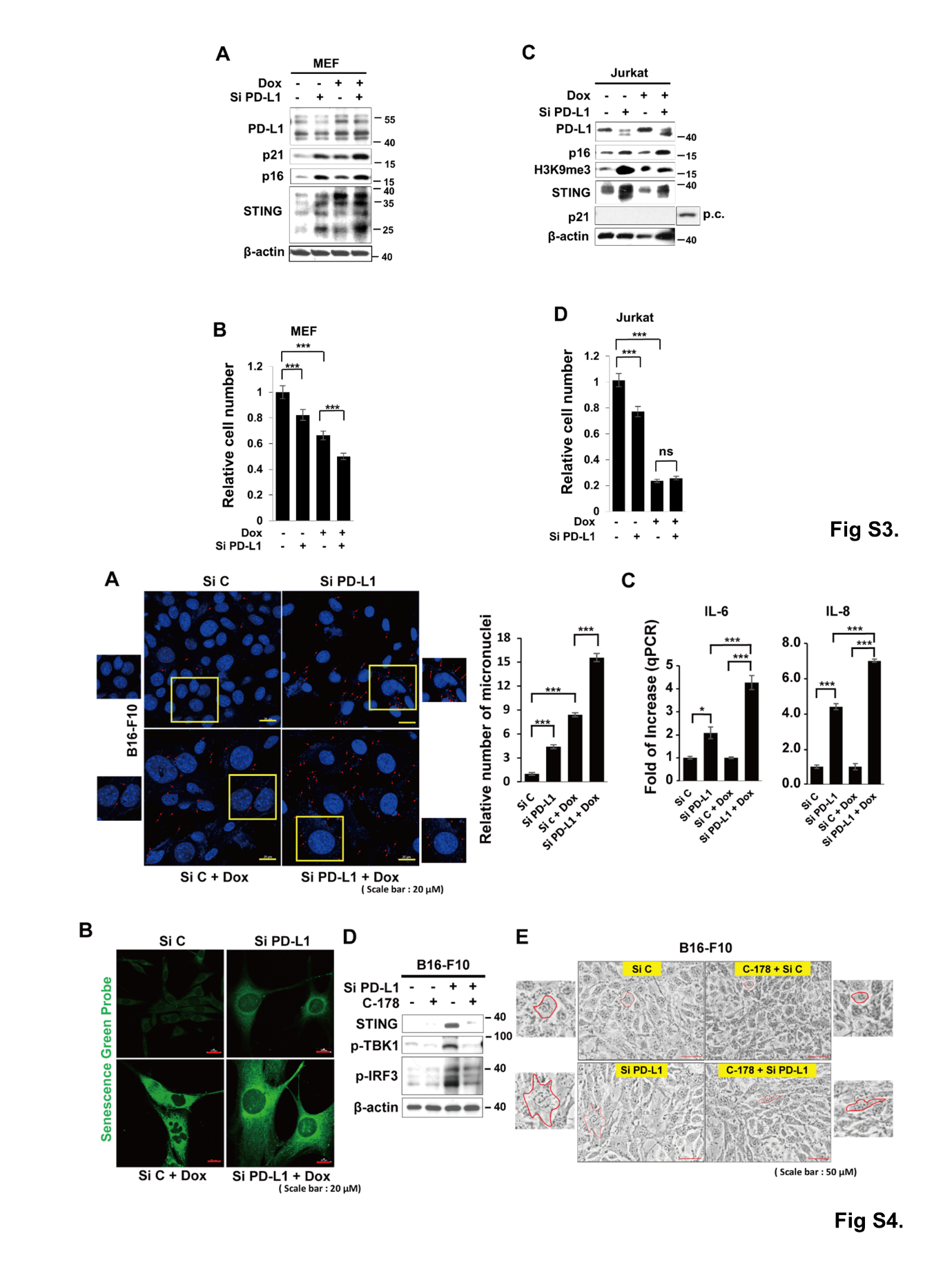


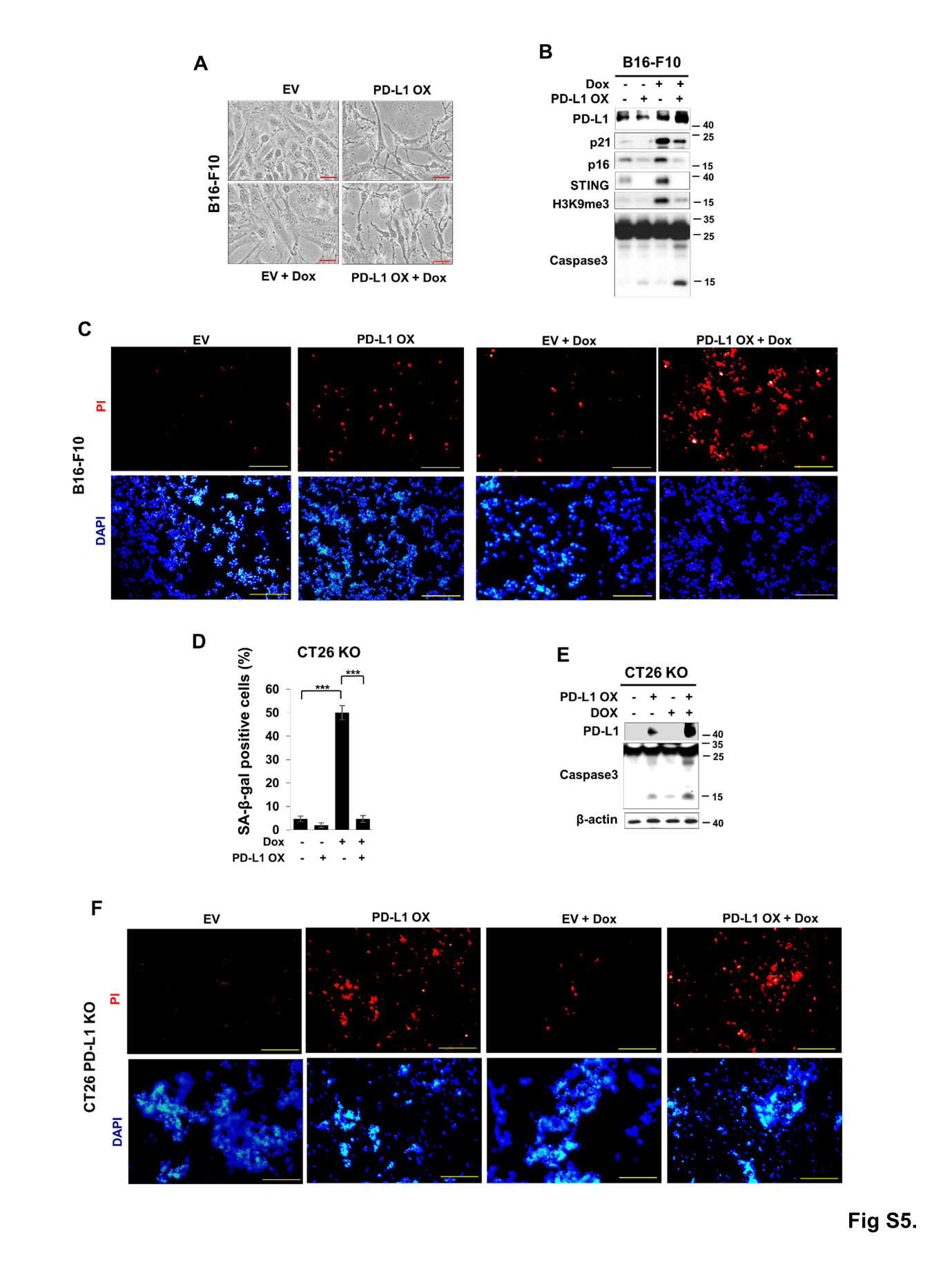


**Supplementary Figure Legends**

**Figure S1. STING mediates PD-L1 targeting-induced senescence.** After obtaining different sequences of PD-L1 siRNA from Figure 1, B16-F10 cells were transfected with 100 nM Control (siC) or PD-L1 (2^nd^ siPD-L1) siRNA prior to 100 ng/mL Dox treatment. Next, the morphological changes (A), SA-β-Gal-positive cell numbers (B, C), and western blot analysis for the indicated proteins (D) were analyzed at day 3 post-Dox treatment. Scale bars = 50 μm. The significance of the statistical difference for among three group was calculated using a one-way analysis of variance and Newman-Keuls. Quantitative data is expressed as the mean ± SD. ***p* < 0.01, ****p* < 0.001.

**Figure S2. STING mediates PD-L1 targeting-induced senescence in human melanoma and lung cancer cells.** Human melanoma cells A375 (A–D), and human lung cancer cells A549 (E, F) were transfected with 100 nM Control (siC) or PD-L1 (siPD-L1) siRNA prior to 100 ng/mL Dox treatment. The morphological changes (A, E), SA-β-Gal positivity (B, C), and western blot analysis for the indicated proteins (D, F) were assessed at day 3 post-Dox treatment. Scale bars = 50 μm. The significance of the statistical difference for among three group was calculated using a one-way analysis of variance and Newman-Keuls. Quantitative data is expressed as the mean ± SD. ****p* < 0.001.

**Figure S3. STING mediates PD-L1 targeting-induced senescence in MEF and human T cell leukemia cells.** Mouse embryonic fibroblast (MEF) cells (A, B) and Jurkat human T lymphocytes (C, D) were transfected with 100 nM Control (Si C) or PD-L1 (SiPD-L1) siRNA prior to 100 ng/mL Dox treatment. Next, western blot analysis was performed for the indicated proteins at day 3 post-Dox treatment (A, C) and relative cell numbers were assessed (B, D). The significance of the statistical difference for among three group was calculated using a one-way analysis of variance and Newman-Keuls. Quantitative data is expressed as the mean ± SD. ns = not significant. ***p* < 0.01, ****p* < 0.001.

**Figure S4. STING inhibition abrogates PD-L1 depletion-induced senescence.**

B16-F10 cells were treated with Si Control or Si PD-L1 1 day before 100 ng/ml Doxorubicin treatment then assessed on day 3 (A-C). (A) Micronuclei were analyzed. The red arrow indicates micronuclei. (B) For SA-B-Gal positive cells, fluorescence signal was captured using the standard FITC settings. Real time-PCR was performed for IL-6 and IL-8. (C). Cells were treated with a pharmacological STING inhibitor (C-178) 2 h prior to treatment with 100 nM Control (siC) or PD-L1 (siPD-L1) siRNA transfection. Western blot assays for the indicated proteins (C) and the morphological changes (D) were analyzed on day 3 post-treatment. Scale bars = 50 μm.

**Figure S5. PD-L1 overexpression abolishes Dox-induced senescence.**

B16-F10 (A–C) and PD-L1 KO CT26 (D–F) cells were transfected with the PD-L1 plasmid 24 h prior to treatment with 100 ng/mL Dox. Then, the cells were imaged (A), and western blot (B) analysis was performed for the indicated proteins. Propidium iodide (PI) was also used for the apoptosis assay (C). Additionally, SA-β-Gal staining (D) and western blot analysis were performed for the indicated proteins (E), and PI staining (F) were performed using PD-L1 KO CT26 cells. Scale bars = 50 μm (A) and 100 μm (C, F). OX: overexpression. The significance of the statistical difference for among three group was calculated using a one-way analysis of variance and Newman-Keuls. Quantitative data is expressed as the mean ± SD. ****p* < 0.001.

**Table S1. List of SiRNA and qPCR primers used in Experiments.**

| **SiRNA** |  |
| --- | --- |
| Si Control | Sense: CCUACGCCACCAAUUUCGU |
|  | Antisense : ACGAAAUUGGUGGCGUAGG |
| Si PD-L1-1^ST^ (m) | Sense: GGUCAACGCCACAGCGAAUUU |
|  | Antisense: AUUCGCUGUGGCGUUGACCUU |
| Si PD-L1-2^ND^ (m) | Sense: GAGGUAAUCUGGACAAACA |
|  | Antisense: UGUUUGUCCAGAUUACCUC |
| Si PD-L1(h) | Sense: AGACGUAAGCAGUGUUGAA |
|  | Antisense: UUCAACACUGCUUACGUCU |
| Si STING (m) | Sense: GGAUCCGAAUGUUCAAUCA |
|  | Antisense : UGAUUGAACAUUCGGAUCC |
| **Chip assay primers** |  |
|  |  |
| STING promoter primer F (m) | TGGTTGTGAACAGCCATGTAG |
| STING promoter primer R (m) | ACCTGAATCCTCCCCGTATC |
