# Supplementary figures and images for: STING mediates nuclear PD-L1 targeting-induced senescence in cancer cells
Source: Cell Death Dis. 2022 Sep 15;13(9):791. doi: 10.1038/s41419-022-05217-6 (PMC9477807; doi:10.1038/s41419-022-05217-6)

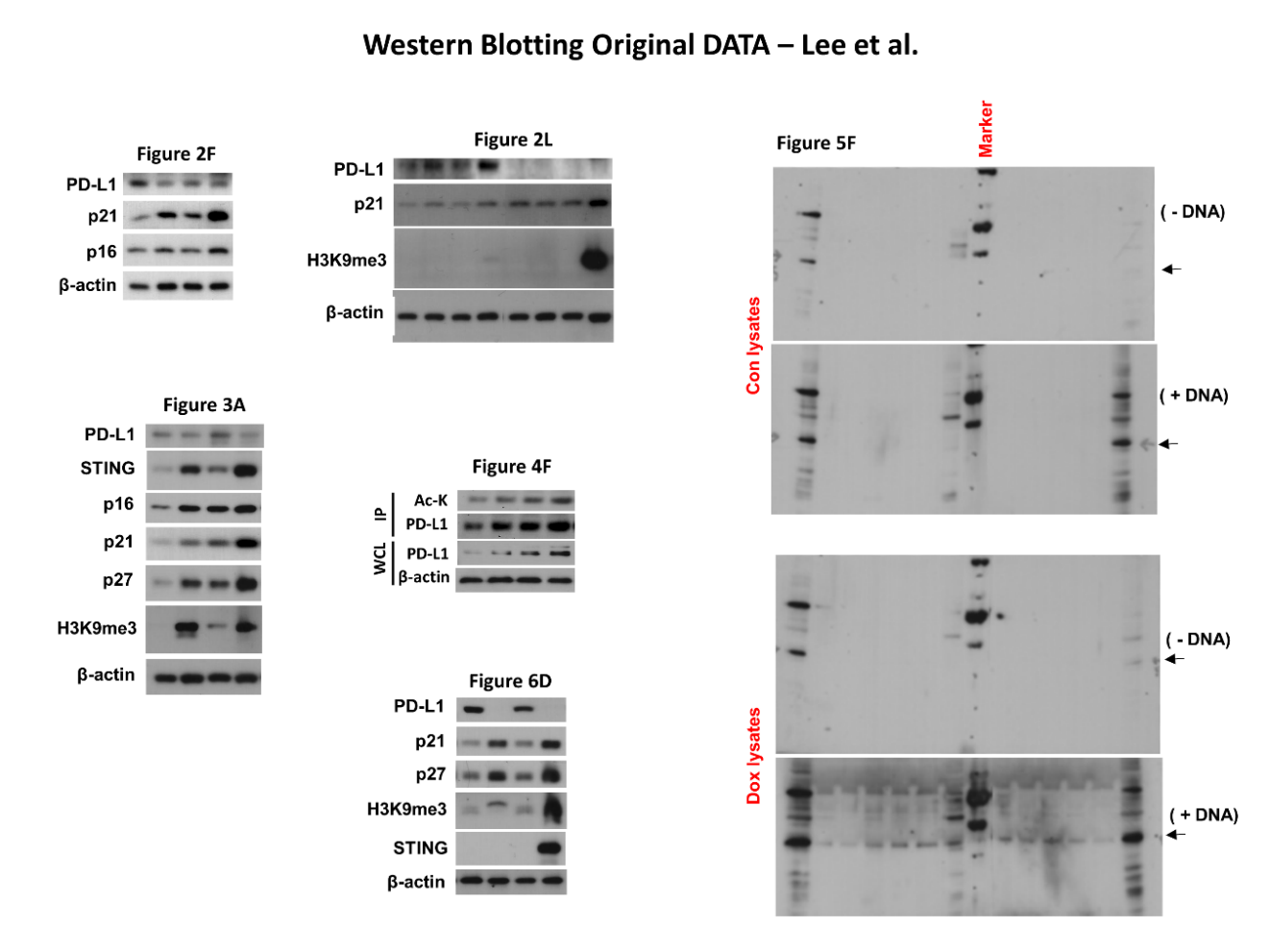


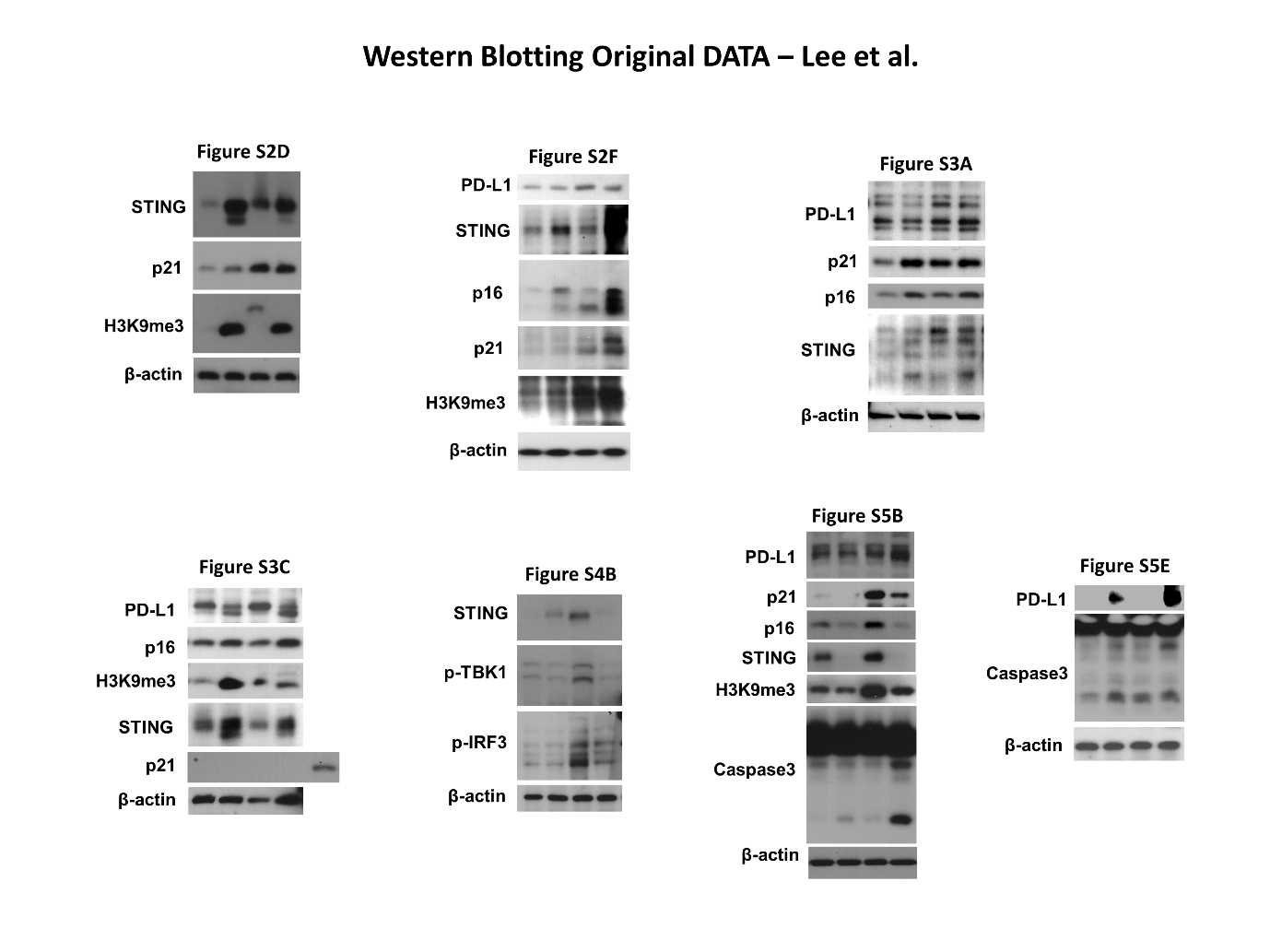

Supplement: Supplementary file 2 — Supplementary Western Blot data [file 41419_2022_5217_MOESM2_ESM.docx]
